# Extract from *Rumex acetosa* L. for Prophylaxis of Periodontitis: Inhibition of Bacterial *In Vitro* Adhesion and of Gingipains of *Porphyromonas gingivalis* by Epicatechin-3-O-(4β→8)-Epicatechin-3-O-Gallate (Procyanidin-B2-Di-Gallate)

**DOI:** 10.1371/journal.pone.0120130

**Published:** 2015-03-24

**Authors:** Jana Schmuch, Sabine Beckert, Simone Brandt, Gesine Löhr, Fabian Hermann, Thomas J. Schmidt, Thomas Beikler, Andreas Hensel

**Affiliations:** 1 University of Münster, Institute for Pharmaceutical Biology and Phytochemistry, Münster, Germany; 2 Heinrich-Heine-University, Department of Operative Dentistry, Periodontics and Endodontics, Düsseldorf, Germany; University of Toronto, CANADA

## Abstract

**Background:**

The aerial parts of *Rumex acetosa* L. have been used in traditional European medicine for inflammatory diseases of the mouth epithelial tissue. The following study aimed to investigate the influence of a proanthocyanidin-enriched extract from *R*. *acetosa* extract against the adhesion of *Porphyromonas gingivalis (P*. *gingivalis)*, a pathogen strongly involved in chronic and aggressive periodontitis. A further goal was to define the bioactive lead structures responsible for a potential antiadhesive activity and to characterize the underlying molecular mechanisms of the antiadhesive effects.

**Methodology:**

An extract of *R*. *acetosa* (RA1) with a defined mixture of flavan-3-ols, oligomeric proanthocyanidins and flavonoids, was used. Its impact on *P*. *gingivalis* adhesion to KB cells was studied by flow cytometry, confocal laser scanning microscopy and *in situ* adhesion assay using murine buccal tissue. RA1 and its compounds **1** to **15** were further investigated for additional effects on gingipain activity, hemagglutination and gene expression by RT-PCR.

**Principal Findings:**

RA1 (5 to 15 μg/mL) reduced *P*. *gingivalis* adhesion in a dose-dependent manner to about 90%. Galloylated proanthocyanidins were confirmed to be responsible for this antiadhesive effect with epicatechin-3-O-gallate-(4β,8)-epicatechin-3’-O-gallate (syn. procyanidin B2-di-gallate) being the lead compound. Ungalloylated flavan-3-ols and oligomeric proanthocyanidins were inactive. RA1 and the galloylated proanthocyanidins strongly interact with the bacterial virulence factor Arg-gingipain, while the corresponding Lys-gingipain was hardly influenced. RA1 inhibited also hemagglutination. *In silico* docking studies indicated that epicatechin-3-O-gallate-(4β,8)-epicatechin-3’-O-gallate interacts with the active side of Arg-gingipain and hemaglutinin from *P*. *gingivalis*; the galloylation of the molecule seems to be responsible for fixation of the ligand to the protein. In conclusion, the proanthocyanidin-enriched extract RA1 and its main active constituent procyanidin B2-di-gallate protect cells from *P*. *gingivalis* infection by inhibiting bacterial adhesion to the host cell. RA1 and procyanidin B2-di-gallate appear to be promising candidates for future cytoprotective preparations for oral mouth care products.

## Introduction

Periodontitis, caused by a polymicrobial colonization of periodontal tissues is clinically characterized by swelling and bleeding of the gums, and leads if left untreated to the destruction of the tooth supporting system and eventually tooth loss. The formation of a complex biofilm is one of the primary etiological factors for periodontitis [1]. Endo- and exogenous products from the bacteria within the biofilm can result in a dysbalanced host immune response by changing the local expression profiles of a variety of chemokines, cytokines and metalloproteinases thus triggering the subsequent inflammation [2].

Although the majority of microorganisms colonizing the gingival sulcus at or below the gingival margin are compatible with periodontal health, a subset of species may cause or contribute to the progression of periodontitis. Around 15 to 20 bacterial species have been found to be closely associated to periodontal disease. Amongst them *Porphyromonas gingivalis (P*. *gingivalis)* and *Aggregatibacter actinomyetemcomitans (A*. *actinomycetemcomitans)* seem to be the most virulent ones [3]. The opportunistic gram-negative anaerobic bacterium *P*. *gingivalis* plays a dominant role in chronic and aggressive forms of periodontitis [4–6]. Furthermore, subgingival colonization with high rates of this bacterium in the infected tissue has been demonstrated to increase the risk of disease progression significantly [7]. Additionally *P*. *gingivalis* has emerged as a potential mediator in the etiology of presumably unrelated chronic diseases, such as rheumatoid arthritis (for review see [8]), cardiovascular diseases, diabetes and, more recently, different types of oral cancers (for review see [9]).

At present, periodontal therapy generally relies on reducing bacterial masses from the infected sites by non-surgical, e.g. supra- and subgingival debridement and surgical procedures [10]. Antibiotics are used to support the therapy, but often fail when exclusively applied because of several pharmacokinetic reasons: low concentrations in the gingival crevice fluids or wash out effects decrease antibiotic activity and also biofilm organization of bacteria can prevent effective antibiotic concentration in an anaerobic environment with low bacterial multiplication rates [11]. Antiinflammatory medication using non-steroidal anti-inflammatory drugs is used in the clinical practice, but a rationalized benefit has not been evaluated finally [12]. Over the past few years plant derived natural products, mainly polyphenols, have been described as putative adjunctive therapy that aims to reduce inflammation as well as interaction with the bacterial adhesion of *P*. *gingivalis* to the host cells (for review see [12]). While flavonoids are mainly involved in the inhibition of inflammatory process (for review see [12]), proanthocyanidins inhibit biofilm formation [13], bacterial adhesion [14], proteolytic activity of pathogens and cytokine production by immune and mucosal cells and matrixmetalloproteinase production [13, 15, 16]. These effects display some strong advantages over the use of antibiotics, since the drug would act at an early stage of the disease process, and is independent from the atmospheric conditions, bacterial multiplication rates and most importantly does not foster antibiotic resistance development.

Development of antiadhesive compounds against *P*. *gingivalis* therefore can be a promising cytoprotective new strategy. The multi-target adhesion of *P*. *gingivalis* to epithelial cells is very complex. Fimbriae mediate adherence to other oral bacterial species, to various host components, such as hemoglobin, collagen, fibronectin and to periodontal cell surface [17, 18]. Additionally, 5 different hemagglutinins, associated with lipopolysaccharides and lipids on the cell surface, and a secreted *exo*-hemagglutinin are responsible for effective binding to erythrocytes and to epithelial cells [19].

The most potent adhesins and virulence factors are the gingipains, three cysteine proteases that bind and cleave a wide range of host proteins [20]. Gingipains are trypsin-like cysteine proteases and are classified into two groups based on substrate specificity. The arginine-specific cysteine protease (Arg-gingipain, Rgp) is encoded by genes *rgp*A and *rgp*B and the lysine-specific cysteine protease (Lys-gingipain, Kgp) is encoded by *kgp* gene. The gingipains are located on the surface of *P*. *gingivalis* from where subfractions are secreted into the extracellular fluid [21]. Due to their proteolytic activity, gingipains are capable of degrading host proteins, such as collagen, fibronectin, immunoglobulin G and TNFα [22]. Due to the adhesin function, the gingipains directly bind to extracellular matrix proteins [23, 24] or indirectly contribute to bacterial adhesion by processing the fimbrillin subunit [25].

It is interesting to recognize that bacterial species exist in defined complexes in subgingival plaques; especially the “red complex”, consisting of *P*. *gingivalis*, *Bacteroides forsythus* and *Treponema denticola* is detected frequently in deeper periodontal pockets [26] in lesions of adult periodontitis [27] and it has been realized that this red complex showed the strongest relationship with the clinical parameters of periodontitis [26]. Additionally it seems interesting that members of this complex cooagregate strongly [28] and may produce growth factors required by another organism in this complex [29]. Therefore potential therapies of periodontitis that affect one of the species of the red complex might influence the colonization of the other species [26].

Due to the complex adhesion strategy of *P*. *gingivalis* a multi target therapy using complex compound mixtures with affinity against the different bacterial adhesins has to be considered; use of a single compound with inhibitory activity against a single adhesin would probably not be sufficient for an effective inhibition of bacterial attachment to the host cells. During the search for potential antiadhesive compounds with affinity to *P*. *gingivalis* fimbriae or OMPs a proanthocanydin-enriched extract from *Rumex acetosa* L. (Polygonaceae) was shown to exhibit antiadhesive activity against *P*. *gingivalis*. The aerial parts of this commonly called sorrel are used traditionally for treatment of skin irritations and diarrhea but have also described for viral infections with Herpes and Influenza virus [30, 31].

**Fig 1:**
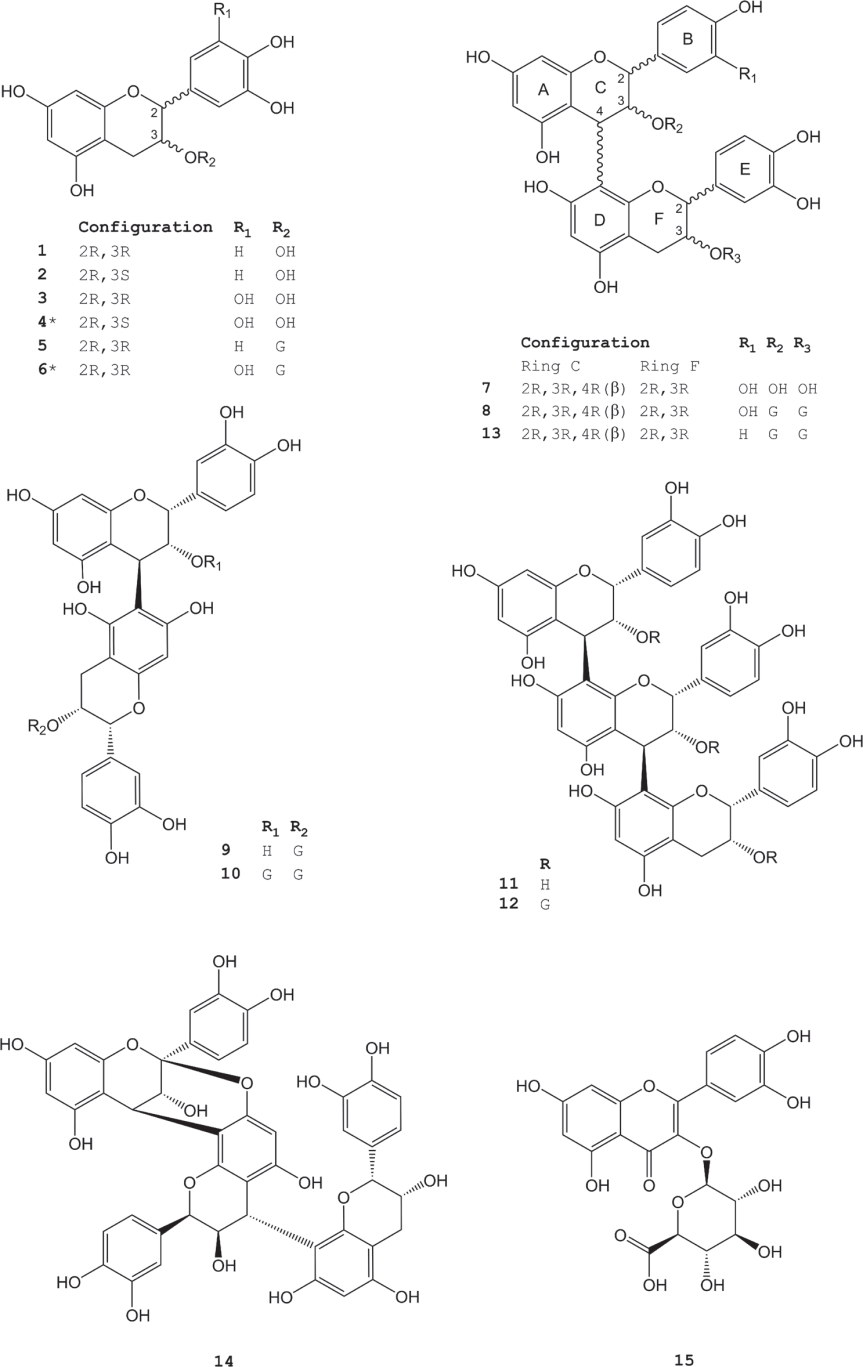
Structural features of flavan-3-ols, proanthocyanidins and quercetin-3-O-glucuronid tested for antiadhesive and ant-gingipain activity against *P*. *gingivalis*; compounds not isolated from *Rumex acetosa* are marked by asterisk and have been used to obtain complete structure-activity relations; G: gallic acid.

Modern phytotherapeutical preparations with nationally registered drug status in Europe contain extracts from *R*. *acetosa* for treatment of acute and chronic infections of the upper respiratory system. Recently, the phytochemical composition of extracts from *R*. *acetosa* has been investigated extensively [32] indicating the presence of monomeric flavan-3-ols, proanthocyanidins and flavonoids. Relevant compounds from *R*. *acetosa* used in this study and characterizing the extract are displayed in Fig. 1.

## Materials and Methods

### Chemicals, reagents, plant material, extract and isolated compounds of Rumex acetosa

If not stated otherwise chemicals and reagents were obtained from VWR (Darmstadt, Germany). Compound **3** was obtained from Sigma (Deisenhofen, Germany) and **6** from ChengduBioPurify Phytochemicals (Bejing, China). Preparation of the *Rumex acetosa* L. extract RA has been described recently [32] using *R*. *acetosa* herbal material (batch 14593) from A. Galke (Gittelde, Germany). In principle the plant material was exhaustively extracted with acetone/water (7:3), followed by evaporation of the organic solvent of the extract *in vacuo*, removal of precipitated chlorophyll by filtration, removal of lipohilic compounds by extraction with heptan and lyophilisation of the aqueous phase to yield the extract RA1 [32]. The isolation and structural characterization of proanthocyanidins and flavonoids (see Fig. 1) from RA was performed as described [32]. In principal RA1 was partitioned between water and ethylacetate. After removal of the solvent, the residues were lyophilized to yield a water-soluble fraction (W) and an EtOAc-soluble fraction (E). E was fractionated by column chromatography on Sephadex LH20 using a step gradient with ethanol, methanol and an acetone-water (7:3) mixture. Fractions obtained were further purified towards the pure compounds by a combination of multilayer countercurrent chromatography (MLCCC), fast centrifugal partition chromatography (FCPC) and low pressure chromatography on MCI gel CHP20P and MPLC on RP18 stationary phase (for details see [24]). Structure elucidation was performed by 1D- and 2D-NMR, circular dicroism (CD), determination of the optical rotation and ESI-MS experiments. Detailed description of the analytical data sets see [32].

### Analytical characterization of RA (UHPLC)

Analytical quality control of RA1was performed by UHPLC by quantitation of the lead compounds epicatechin-3-O-gallate **5**, epicatechin-(4β→8)-epicatechin (syn. procyanidin B2) **7**, epicatechin-3-O-gallate-(4β→8)-epicatechin-3-O-gallate (syn. procyanidin B2-di-gallate) **8** epicatechin-(β→8,2β→O→7)-epicatechin-(4β→8)-epicatechin (syn. cinnamtannin B1) **14**, quercetin-3-O-glucuronide **15**.

1 mg of RA was dissolved in 1 mL of acetonitrile/0.1% TFA in water (1:1), containing 0.1 mg/mL ascorbic acid. After centrifugation at 1000 × g / 5 min the supernatant was transferred into a vial and the test solution was analyzed by UPLC.

Instrumentation: Acquity UPLC Class, Binary Solvent Manager, Column Manager, Column oven (25°C), Sample Manager FTN, PDA λe Detector, software Empower 3 2010 (Waters, Milford, USA). Stationary phase: Acquity UPLC BEH Shield RP 18, 1.7 μm, 2.1 × 100 mm (Waters, Milwaukee, U.S.A.). Mobile phase: A water containing 0.1% TFA, B acetonitrile; gradient: t_0min_ 92% A, t_17min_ 65% A, t_19_ min 0% A, t_21min_ 100% A, t_22min_ 92% A. Detection: DAD λ 210–500 nm. Injection volume: 1 μL. Flow: 0.4 mL/min,

### *P*. *gingivalis* strains and growth conditions

*P*. *gingivalis* (ATCC 33277) was cultured under anaerobic conditions (Anaerocult, Merck, Darmstadt, Germany) at 37°C on solid sheep blood agar (per 1 L: 16 g agar, 15 g trypticase peptone, 5 g neutralized soja peptone, 5 g sodium chloride, 5 g yeast extract, 0.5 g L-cysteine, 50 ml sheep blood) enriched with 10 mg vitamin K and 5 mg hemin. For quality control purposes (identity and purity), every third passage of the bacterium was subjected to quantitative *fimA* gene PCR analysis. For assays an overnight liquid culture in the exponential growth phase was used, prepared from agar grown *P*. *gingivalis* and suspended in liquid culture medium at an OD_660_ 0.1.To obtain bacteria in the exponential phase, the liquid culture was incubated under anaerobic conditions for 24 h. OD_660_ 0.05 was correlated to 0.32 × 10^8^ CFU/mL.

The clinical isolate PG3 was obtained from a patient with a moderate form of generalized, chronic periodontitis from University Hospital Münster (Germany).

### Cell culture

KB cells (ATCC CCL-17), derived from a human oral epidermoid carcinoma, were kindly provided by Dr. S. Eick (University of Jena, Germany). Recent testing performed during ATCC accessioning showed that this line may be contaminated with HeLa cells. This finding is based on isoenzyme analysis, HeLa marker chromosomes and DNA fingerprinting (see www.atcc.org). KB cells have been widely used in studies with microorganisms related to periodontitis, especially with *P*. *gingivalis*. Especially the data on invasion of *P*. *gingivlias* contributed to the choice of this cell line for the majority of these studies. While it is recognized that expression of KB cell surface receptors for *P*. *gingivalis* may differ from that of non-transformed cells [33] significant similarities to human gingival epithelial cells have also been reported [34]. A comparative study on nuclear targeting of *P*. *gingivalis* proteases between KB cells, HeLa cells and SCC4 cells did not show any relevant differences for the nuclear entry of HRgpA into the different cell lines of both oral and non-oral origin [35]. From these data and the manifold publications on *P*. *gingivalis* and KB host cells we have the feeling that the test model should be reliable and valid.

KB cells were cultured in Earl`s minimum essential medium (EMEM) (Lonza, Basel, Switzerland), supplemented with 8% (v/v) heat-inactivated FCS (PAA Laboratories, Cölbe, Germany) and 50 μg/ml gentamicin (MP Biomedicals, Irvine, USA) at 5% CO_2_ / 37°C. Passaging was performed twice a week at 80–100% confluence to a maximum of 15 passages.

### Cytotoxicity assay

Cell proliferation was monitored by using BrdU Cell Proliferation ELISA kit (Roche, Pensberg, Germany) with 3 × 10^4^ cells in 96 well plates and 24 h incubation time with test compounds, dissolved in EMEM. EMEM supplemented with 0.1% FCS served as positive control. Mitochondrial activity was determined by MTT assay using 1 × 10^5^ cells per well and 6 h incubation with test compounds [36].

### Antibacterial susceptibility test

For disk diffusion test a 3-day-old *P*. *gingivalis* culture was harvested and suspended in sterile PBS. The optical density was adjusted to OD_660_ 0.2 and 100 μL of the suspension were plated on blood agar. Paper disks (BD Sensi-Disc, 6mm, BD Heidelberg, Germany) with 20 μL of RA1 (1 to 100 μg/mL in PBS, corresponding to 0.02 to 200 μg per disk) were placed circularly in equal distances on the inoculated plate. Prior to anaerobic incubation at 5% CO_2_ / 37°C, plates were incubated for 10 minutes side up at RT, to ensure the diffusion of the test compounds into the agar. Sterile PBS was used as negative and amoxicillin as positive control. After an anaerobic incubation of 5 days, inhibition zones were analysed.

For investigation of antibacterial influence of the test compounds against *P*. *gingivalis* in broth culture an overnight liquid culture of *P*. *gingivalis* was adjusted to an optical density of OD_660_ 0.2. RA1 at 50 and 100 μg/mL, dissolved in PBS and bacteria were incubated anaerobically and the growth was monitored by determination of OD_660_. In addition to that, 100 μL of bacterial suspension were plated out on agar plates after 24 h, to control the number. For the preparation of test solutions a stock solution (1 mg/mL) was prepared by adding 10 μl of DMSO to 1 mg of the test compound. The resulting solution was diluted to 1000 μL in PBS.

For the preparation of test solutions a stock solution (1 mg/mL) was prepared by adding 10 μl of DMSO to 1 mg of the test compound. The resulting solution was diluted to 1000 μL.

### Adhesion assay with *P*. *gingivalis* and KB cells

5 × 10^5^ KB cells were seeded in a six well plate and were incubated for 24 h at 37°C and 5% CO_2_ in culture media until a confluence of 80% was achieved. Cells were washed with 2 mL PBS and subsequently incubated in antibiotic- and serum-free medium. Liquid-culture grown *P*. *gingivalis* (24 h, 37°C) were harvested and suspended in 0.5 M NaHCO_3_, pH 8.0. After adjusting the OD_660_ to 0.4, fluorescein isothiocyanate FITC (50 μg/ml) (Sigma-Aldrich, Steinheim, Germany) was added for labelling of the bacteria followed by incubation for 30 min / 37°C. Bacteria were sedimented (3.600 × g, 5 min) and washed twice in PBS to remove unbound FITC. FITC-labelled bacteria were suspended in EMEM. Labelling efficiency was analyzed by fluorescence microscopy.

To investigate the effect of RA on the adhesion of *P*. *gingivalis* on KB cells, various incubation procedures were applied.

- For *preincubation of labelled bacteria*, fluorescence-labeled bacteria (OD_660_ 1.0) were preincubated with test compounds for 30 min at 37°C. After sedimentation and washing with PBS for removal of test compounds, bacteria were resuspended in EMEM and added to KB cells (BCR 100:1) for 90 min at 37°C.
- For *coincubation*, test compounds and fluorescence-labeled *P*. *gingivalis* were simultaneously added to KB cells (BCR 100:1) and incubated for 90 min at 37°C.

TLCK (AppliChem, Darmstadt, Germany) (1 mM) served as positive control. TLCK (Nα-tosyl-L-lysine chloromethyl ketone hydrochloride) represents a specific serine protease inhibitor [37] which irreversibly inhibits trypsin and trypsin-like serine proteases by alkylation of a histidine-46 residue in the active site of the enzyme [38, 39].

After each incubation variant, KB cells were washed three times with 2 mL PBS, detached from the wells by incubation with trypsin-EDTA for 3 min at 37°C, suspended in 2 mL antibiotic- and serum free medium and centrifuged at 400 × g for 3 minutes. The adhesion intensity of FITC-labelled *P*. *gingivalis* on KB cells was immediately analyzed by flow cytometry (FACS Calibur, Becton Dickinson, Heidelberg, Germany). For each sample a pre-measurement phase of 5000 events was performed, until 10000 events of each sample were analyzed. To exclude measurement of cell agglomerates and cell debris, a gated region in the sideward/forward scatter plot was set. Only cells in this gate were counted. Instrument settings were as follows: FCS (Detector): E-1 (Voltage), 3.07 (Amp Gain), Lin (Mode); SSC: 332, 1.00, Lin; FL1: 360, 1.00, Log; FL2: 350, 1.00, Log; FL3: 570, 1.00, Log; FL2-A:-, 1.00, Lin; FL4: 568, -, Log. The relative adhesion was determined by distributing the cell populations in two gates: Gate M1 includes the population of KB cells without adherent bacteria. M2 gates KB cells with adherent bacteria. The relative adhesion of an extract or a compound was determined by using the following equation with A being the text compound and UC the untreated control:

$$\mathrm{relative adhesion}\;[\%]=\frac{\mathrm{Median (FL1)A}\times \mathrm{P (M2)A}}{\mathrm{Median (FL1)UC}\times \mathrm{P (M2)UC}}\times 100$$

### Confocal laser scanning microscopy

KB cells (1.5 × 10^5^) were seeded on glass slides inside removable flexiPERM chambers (Greiner Bio-one, Frickenhausen, Germany). After 48 h at 37°C / 5% CO_2_ 80% confluence was reached. Endosomes were stained with DextranTexasRed (100 μg/mL) 12 h before cells were incubated with *P*. *gingivalis*.

FITC-labeled bacteria alone or together with RA1 were coincubated for 90 minutes with the KB cells at a BCR of 100:1. Unbound bacteria were removed by 3 × washing with 300 μL of PBS. Cells were fixed with 300 μL 3.7% formaldehyde in PBS for 15 min at room temperature. The cells were washed twice with 300 μL of PBS, treated with 300 μL of 0.5% Triton-X100 (Fluka AG, Buchs, Switzerland) in PBS for 10 min and were then washed twice with PBS. To block unspecific binding 300 μL of 3% BSA in PBS were added for 20 min. After 2 × washing with PBS nuclei were stained with DAPI (Sigma-Aldrich, Steinheim; 1 μg/mL, 30 min). The flexiPERM chamber was removed, three drops of Dako fluorescent mounting (Dako, Carpinteria, USA), were added and cells were covered with a coverslip, which was fixed with nail polish. CLSM was performed by a Leica TCS SP2 fluorescence microscope (Leica, Solms, Germany). λ DAPI 405/420–480 nm, λ FITC 488/500–580 nm, λ TexasRed 594/620–700 nm.

### Adhesion assay of *P*. *gingivalis* to murine oral buccal mucosa

The *in situ* adhesion assay with histological murine oral mucosa tissue sections was performed according to [40]. Use of isolated organs from mice corresponded to the German Law for Animal Testing (acceptance No. 84.-02.05.20.12.256). Freshly prepared oral buccal mucosa was obtained from C57Bl/6N mice (Charles River, Sulzfeld, Germany), age 3 months to 1 year, fed with dry feed and water *ad libitum* and kept in standardized housing in 12 h night-day-cycle. Oral buccal mucosa was stored in 4% buffered formalin solution, until it was embedded in paraffin for preparation of tissue sections (3 mm thickness). The preparation of tissue sections was kindly performed by the Institute of Physiological Chemistry and Pathobiochemistry, Münster.

Prior to incubation with fluorescence labeled *P*. *gingivalis*, tissue sections were deparaffinised in a series of xylene (30 min), isopropanol 100% (2 × 2 min), isopropanol 96% (2 × 2 min), isopropanol 70% (2 × 2 min), water (2 min), PBS (2 min) and blocking buffer (20 min).

200 μL of a suspension of FITC-labeled *P*. *gingivalis* (OD_660_ = 1), pretreated with RA1 (100 μg/mL) or TLCK (5 mM) for 90 min, were transferred to deparaffinized histological sections from murine oral buccal mucosa. Incubation was performed for 1 h at RT. Tissue sections were washed 10 times with PBS and analyzed immediately by fluorescence microscopy.

### Gingipain assay [41]

Bacteria from a three-day culture on blood agar plates were suspended in buffer (200 mM Tris Cl, pH 7.6 containing NaCl 150 mM, CaCl_2_ 5 mM, NaN_3_0.02%, Cystein HCl 20 mM) to an OD_660_ of 0.5.

50 μL of this suspension, for measurement of the Lys-gingipain activity, and 10 μL, for determination of the Arg-gingipain activity, were mixed with the respective test solutions (140 or 150 μL). Leupeptin (10 μM) served as positive control. The assay mixture was incubated for 10 min at 37°C. 10 μL of N_α_-Acetyl-L-lysine-4-nitroanilide HCl (4 μg/μL in DMSO) or N_α_-Benzoyl-L-arginine-4-nitroanilide HCl (3 μg/μL in DMSO) respectively were added and a continuous measurement of the absorption at λ 405 nm over a period of 15 min was performed (Tecan-Reader Sunrise). Wells without substrate addition provided the corresponding blank values. Protease activities were related to the untreated control.

### Hemagglutination assay with *P*. *gingivalis*

Colonies of solid medium-grown *P*. *gingivalis* (3- to 5-day cultures) were harvested and suspended in PBS, pH 7.4. 150 μL of the bacterial suspension (OD_660_ 1.0) were mixed with 150 μl MF-containing solution and incubated at 37°C / 30 min. Bacteria were washed (6.000 × g, 5 min), resuspended and serially diluted in 96-well round bottom microtiter plates with PBS. A 50 μl aliquot of each dilution was mixed with 50 μL of human erythrocyte suspension (2% in PBS) and incubated for 3 h at RT. Assays were carried out in duplicate and were repeated at least three times in independent experiments. Evaluation was performed by using an UV reader (Sunrise, Tecan Austria GmbH, Salzburg, Austria) and MicroWin software.

### Gene expression analysis of *P*. *gingivalis* (RT PCR)

Solid medium-grown *P*. *gingivalis* from a 3 days culture were harvested and used to inoculate liquid media (OD_660_ = 0.05). After 24 h bacteria were transferred to 4 mL liquid culture media (OD_660_ = 0.1). RA1 (100 μg/mL) was added. Liquid culture with *P*. *gingivalis* served as negative control. After incubation for 6 h at 37°C bacteria were harvested by centrifugation (11.000 × g, 5 min) and washed with PBS. The pellet was resuspended in PBS (100 μL) and mixed with TE-Buffer (500 μL), 1% SDS, phenol and lysing matrix B (0.4 g) (MP Biomedicals, Irvine, USA). Cells were lysed by FastPrep-24-System (MP Biomedicals, Irvine, USA) (2 × 50 sec, 5.0 m/s^2^) and RNA-isolation was followed by phenol-chloroform-extraction and ethanol-precipitation. Removal of DNA was performed by NucleoSpinrDNase (Machery-Nagel, Düren, Germany) according to the manufacturer`s instructions. Total RNA concentration in each sample was calculated from OD_260_. cDNA was synthesized from 1 μg of total RNA by using the QuantiTectReverse Transcription System (Qiagen, Hilden, Germany). RT-PCR was performed with 2 μL (100 ng) cDNA, 10 μL 2 × QuantiTect Probe PCR Master Mix (Qiagen, Hilden, Germany), 1 μL 20 × Primer Mix, 1 μL 20 × QuantiProbe Solution and 6 μL RNase free water. The oligonucleotide primers and probes [14] were synthesized by Qiagen (Düsseldorf, Germany).

PCR was performed on a 7300 Real Time PCR System (Applied Biosystems (ABI), Germany) and 7300 System Sequence Detection Software (Version 1.2.3, ABI, Germany). A total of 45 cycles were performed after 15 min at 95°C for enzyme activation. Elongation at 76°C / 30 sec, denaturation at 94°C / 15 sec, annealing at 56°C / 30 sec. mRNA levels were expressed as RQ-values, related to the C_T_-value of the 16S rRNA and to the untreated control.

### *In silico* protein-ligand docking

For *in silico* analyses the gingipain RgpB (protein data base ID ICVR was used [42]. Test compounds (Fig. 1) were docked into the active side of Arg-gingipain of *P*. *gingivalis in silico* by the software Molecular Operating Environment (MOE) version 2011.10 (Chemical Computing Group, Montreal, Canada) using the MMFF94x force field as implemented in MOE. The flexible docking method (induced fit, i.e. both the ligand and the protein binding site were treated as flexible) was applied. The docking pose represents the most favorable geometry (lowest calculated S-score) of all investigated compounds.

For *in silico* evaluation of the results obtained in the adhesion assay a fragment of Lys-Gingipain containing the hemagglutinin/adhesin (HA) domain (protein database ID “3M1H”) was employed. The active site of the fragment was identified according to [43] (Arg 1557) and the site finder algorithm implemented in MOE. Compounds, displayed in Fig. 1 were docked into this active site employing the MMFF94x forcefield and the induced fit method supplied by the software MOE (induced fit, i.e. both the ligand and protein binding site were treated as flexible).

### Statistical analysis

Data represent the means ± SD of at least three independent experiments. Statistical analysis for experiments with n ≥ 3 was performed by one way analysis of variance (one way ANOVA) and t-test or Turkey’s Multiple Comparison test respectively with GraphPad Prism 2.01, GraphPad Software, INC., La Jolla, CA, USA. *p* < 0.05 was regarded as statistical significant (*), *p* < 0.01 as highly significant (**) and *p* < 0.001 as very high significant (***).

## Results

### Preparation and characterization of *R*. *acetosa* extract RA1

**Fig 2:**
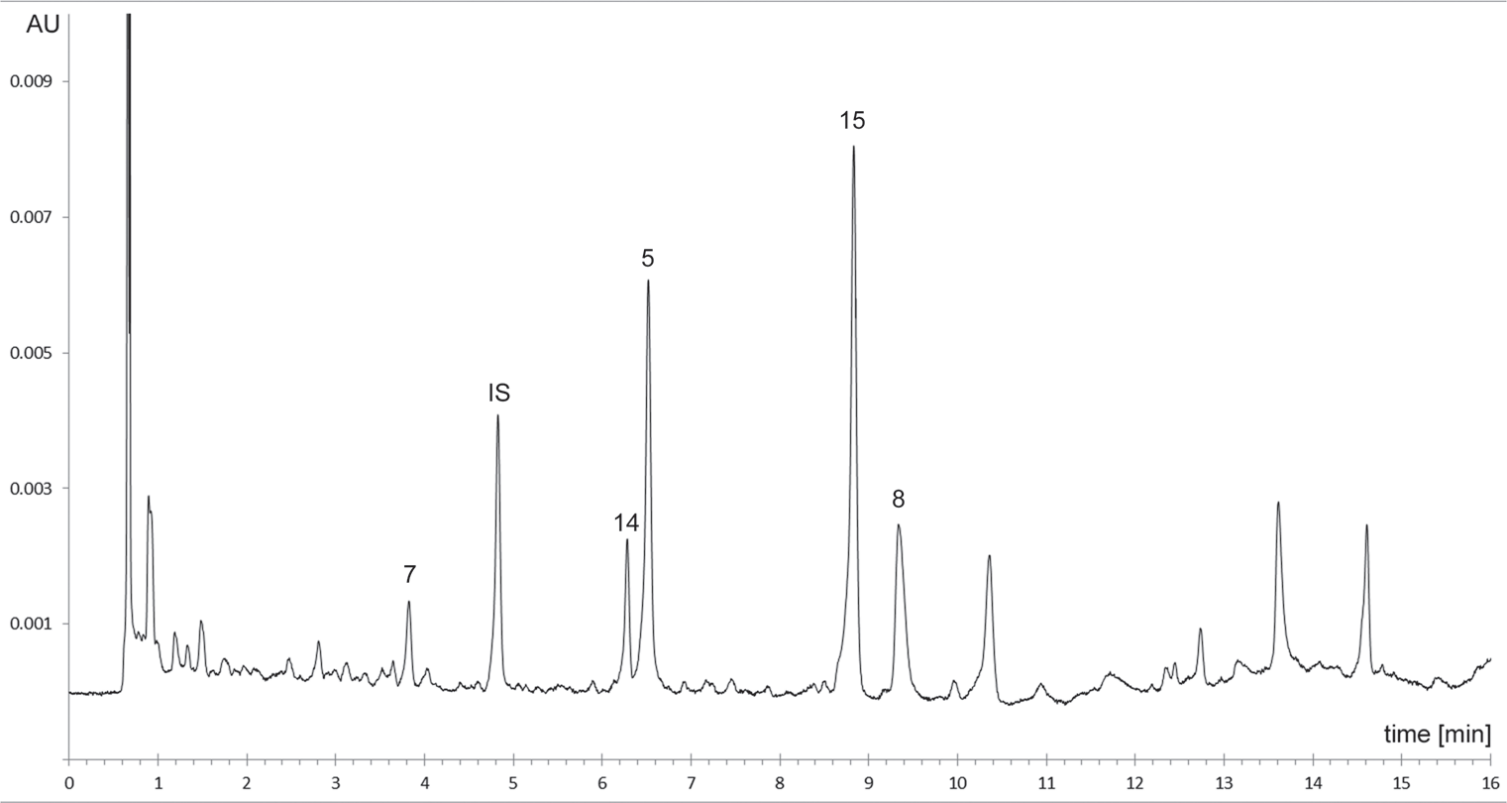
UHPLC of RA1 (1 mg/mL). **7** epicatechin-(4β→8)-epicatechin, **14** epicatechin-(4β-→8)-epicatechin(4β→8,2β→O→7)-epicatechin, **5** epicatechin-3-O-gallate, **15** quercetin-3-O-glucuronide, **8** epicatechin-3-O-gallate-(4β→8)-epicatechin-3-O-gallate, **IS** internal standard epigallocatechin-3-O-gallate.

From the aerial parts of *R*. *acetosa* extract RA1 was prepared (yield 9.3%, related to the starting material) which contains flavonoids, flavan-3-ols and oligomeric procyanidins [32]. For analytical quality control a fast and efficient UPLC method was developed to identify the lead compounds epicatechin-3-O-gallate **5**, epicatechin-(4β→8)-epicatechin **7** (syn. procyanidin B2), epicatechin-3-O-gallate-(4β→8)-epicatechin-3-O-gallate **8** (syn. procyanidin-B2-di-gallate), epicatechin-(4β→8,2β→O→7)-epicatechin-(4β→8)-epicatechin **14** and quercetin-3-O-glucuronide **15**. Fig. 2 displays a representative chromatogram of RA1.

The UPLC method was validated concerning the quantitation of procyanidin-B2-di-gallate **3** according an ICH-compliant protocol [44] indicating sufficient specifity, linearity in the range of 3 to 50 μg/g, accuracy (recovery) of 100.0%, system precision of ± 1.5%, and intermediate precision of ± 1.7%. Using this validated method quantification of epicatechin-3-O-gallate-(4β→8)-epicatechin-3-O-gallate **3** by external standard calibration revealed a content of 11.9 mg/g RA1. The content of compound **1** was determined with 17.9 mg/g, for compound **2** 3.8 mg/g, for compound **4** 6.0 mg/g, and 24.0 mg/g for compound **5**, calculated as procyanidin-B2-di-gallate.

### RA1 inhibits adhesion of *P*. *gingivalis* to KB cells

Preexperiments concerning the influence of RA1 on cell physiology of epithelial and bacterial cells indicated that the extract reduced cell proliferation (BrdU incorporation ELISA) of KB cells after a 24 h incubation at concentrations > 50 μg/mL with an IC_50_ of 227.5 μg/mL.

RA1 up to 100 μg/mL over 5 days incubation did not influence bacterial growth of *P*. *gingivalis* within disk diffusion (data not shown). Also in liquid culture no significant inhibition of bacterial proliferation by RA1 (50 and 100 μg/mL) was observed over a 24 h incubation period (data not shown).

The influence of RA1 on the adhesion of *P*. *gingivalis* to KB cells was analyzed quantitatively by a flow cytometric assay. In principle, FITC-labelled bacteria were coincubated with a monolayer of KB cells. Non-adhering bacteria were removed by washing. After trypsin treatment suspended cells with adhering or invaded labelled bacteria were quantified by FACS. The specific serine protease inhibitor Nα-tosyl-L-lysine chloromethyl ketone hydrochloride (TLCK) served as positive control [45].

**Fig 3:**
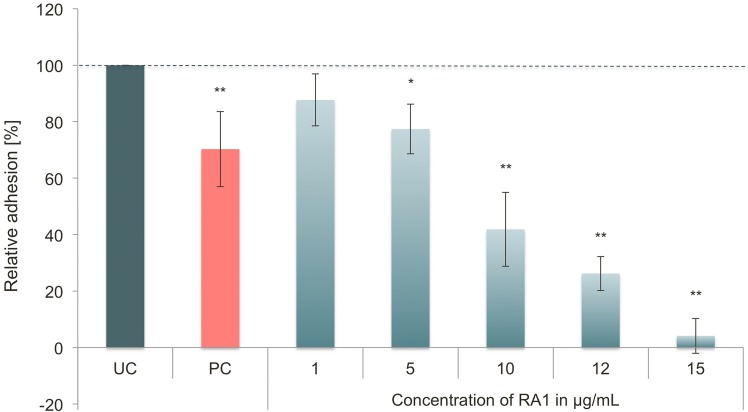
Influence of RA1 on the relative adhesion of FITC-labeled *P*. *gingivalis* to KB cells in the coincubation assay (90 min), determined by flow cytometric analysis. Results are related to the untreated control (UC). The protease inhibitor Nα-tosyl-L-lysine chloromethyl ketone hydrochloride (TLCK, 1 mM) served as positive control (PC). Values represent the mean ± SD from three independent experiments; * p < 0.05; **: p < 0.01.

Cotreament of *P*. *gingivalis* (ATCC 33277) and KB cells with RA1 evoked concentration-dependent inhibition of bacterial adhesion (Fig. 3) with an IC _50_ of 10.8 μg/mL. Interestingly, pretreatment of bacteria with RA1 was much less effective than the cotreatment (30% inhibition at 100 μg/mL).

RA1 at 10 μg/mL was also active against a clinical isolate (PG3) of *P*. *gingivalis*; the relative adhesion of the strain PG3 to KB cells (which was in the untreated control groups twice as high compared to ATCC3277 lab strain!) was inhibited by the extract by more than 90% (data not shown).

**Fig 4:**
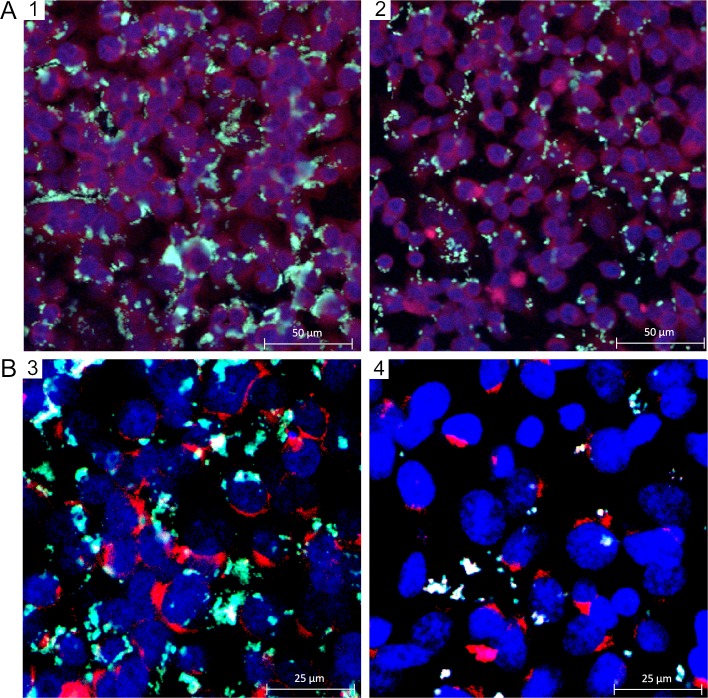
Representative confocal laser scanning microscopy images of FITC labeled *P*. *gingivalis* during different incubation protocols with KB cells. (A) Coincubation (90 min) of KB cells with FITC-labeled *P*. *gingivalis* (BCR 100:1) and RA1 (10 μg/mL); 1: untreated control 2: RA1 (10 μg/mL). (B) Preincubation of KB cells with RA1 (10 μg/mL) for 12 h and subsequent infection with *P*. *gingivalis* (BCR 100:1) for 90 min; 3: untreated control; 4: RA1 (10 μg/mL). Bacteria are stained with FITC (green), nuclei of KB cells with DAPI (blue) and endosomes with Dextran Texas Red (red).

Confocal laser scanning microscopy was used for visualization of the potential antiadhesive effect of RA1 by staining nuclei of KB cells with DAPI, endosomes with Dextran Texas Red and bacteria with FITC. While strong bacterial adhesion to KB cells got obvious in the non-treated control group, considerably decreased bacterial binding of *P*. *gingivalis* was observed for cell groups treated with RA1 (10 μg/mL) (Fig. 4A). Internalisation of *P*. *gingivalis* into the cells was not observed under the chosen conditions. Preincubation of KB cells with RA1 (10 μg/mL) for 12 h and subsequent infection with *P*. *gingivalis* for 90 min showed a pronounced antiadhesive effect of the extract (Fig. 4B).

**Fig 5:**
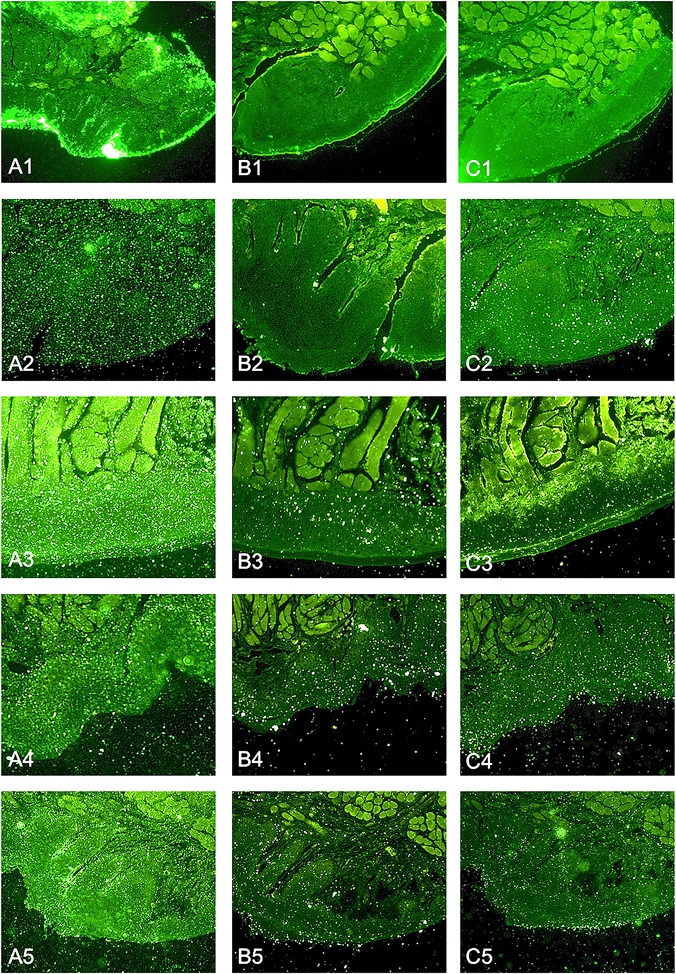
Representative fluorescence microscopy images of FITC labeled *P*. *gingivalis* adherent to murine oral mucosa sections from four independent experiments; data sets 4 and 5 originate as technical replicates from the same experiment to indicate intraassay reproducibility A 1–5: untreated control B 1–5: positive control, pretreated with 5 mM TLCK for 90 minutes C 1–5: RA1 100 μg/mL (preincubation of bacteria for 90 minutes). Magnification: 100 ×. Images are equalized in brightness, contrast and fluorescence intensity.

For confirmation of these results an *in situ* assay on murine buccal mucosa sections was performed based on a protocol of [40, 46]. Therefor bacteria were pretreated with test samples (only medium for untreated control group, TLCK (5 mM) for positive control, RA1 (100 μg/mL) for 90 minutes. Pretreated and FITC-labeled bacteria were subsequently incubated with histological oral mucosa sections from mice buccal tissue for 1 h. The adhesion of FITC labeled bacteria was evaluated by fluorescence microscopy. Fig. 5 shows representative results of four independent adhesion experiments indicating diminished bacterial adhesion in the TLCK and RA1 treated groups.

### Galloylated oligomeric proanthocyanidins are responsible for antiadhesive activity

For pinpointing which compound from the complex extract RA1 is responsible for this antiadhesive effect the flavan-3ols, proanthocyanidins and quercetin-3-O-glucuronide (Fig. 1) were isolated on a preparative scale [32] and investigated concerning the respective influence on bacterial adhesion. To complete structure-activity testing additionally epigallocatechin-3-O-gallate **5** was inserted into the assays, a compound similar to flavan-3-ols from RA1, but not present in the extract. Table 1 displays the results, indicating that galloylation is a prerequisite for antiadhesive flavan-3-ols (e.g. **1**, **2**, **3** inactive but **5** active). Additionally tri-hydroxylation of the B-ring of the galloylated flavan-3-ol (e.g. **6**) will further increase the antiadhesive activity. Di- and trimerisation of galloylated flavan-3-ols forms highly active compounds **8** and **12**, while the respective ungalloylated oligomers **7** and **11** do not exert any activity. From these data we deduce procyanidin-B2-3-O-digallate **8** as lead compound for the antiadhesive activity of RA1 which exerts a high activity and is present at higher concentrations in RA1. In contrast compound **12** is even more active, but only present in traces in RA1.

**Table 1:** Influence of flavan-3-ols and proanthocyanidins from RA1 on the adhesion of *P*. *gingivalis* to KB cells in the coincubation adhesion assay.

Test compound	1	3	4	5	6	7	8	12	14	15
IC_**50**_ [μM]	>50	>50	>50	>50	**38.0**	>50	**19.6**	**3.8**	**19.8**	>100
IC_**75**_[μM]				**30.0**						

Data represent IC_50_ resp. IC_75_ for compound 5. IC_50_: concentration of the test compound reducing the bacterial adhesion to 50%, related to the untreated control group; IC_75_: concentration of the test compound 5 reducing the bacterial adhesion to 75%, related to the untreated control group.

### RA1 and galloylated proanthocyanidins inhibit Arg-gingipain

**Fig 6:**
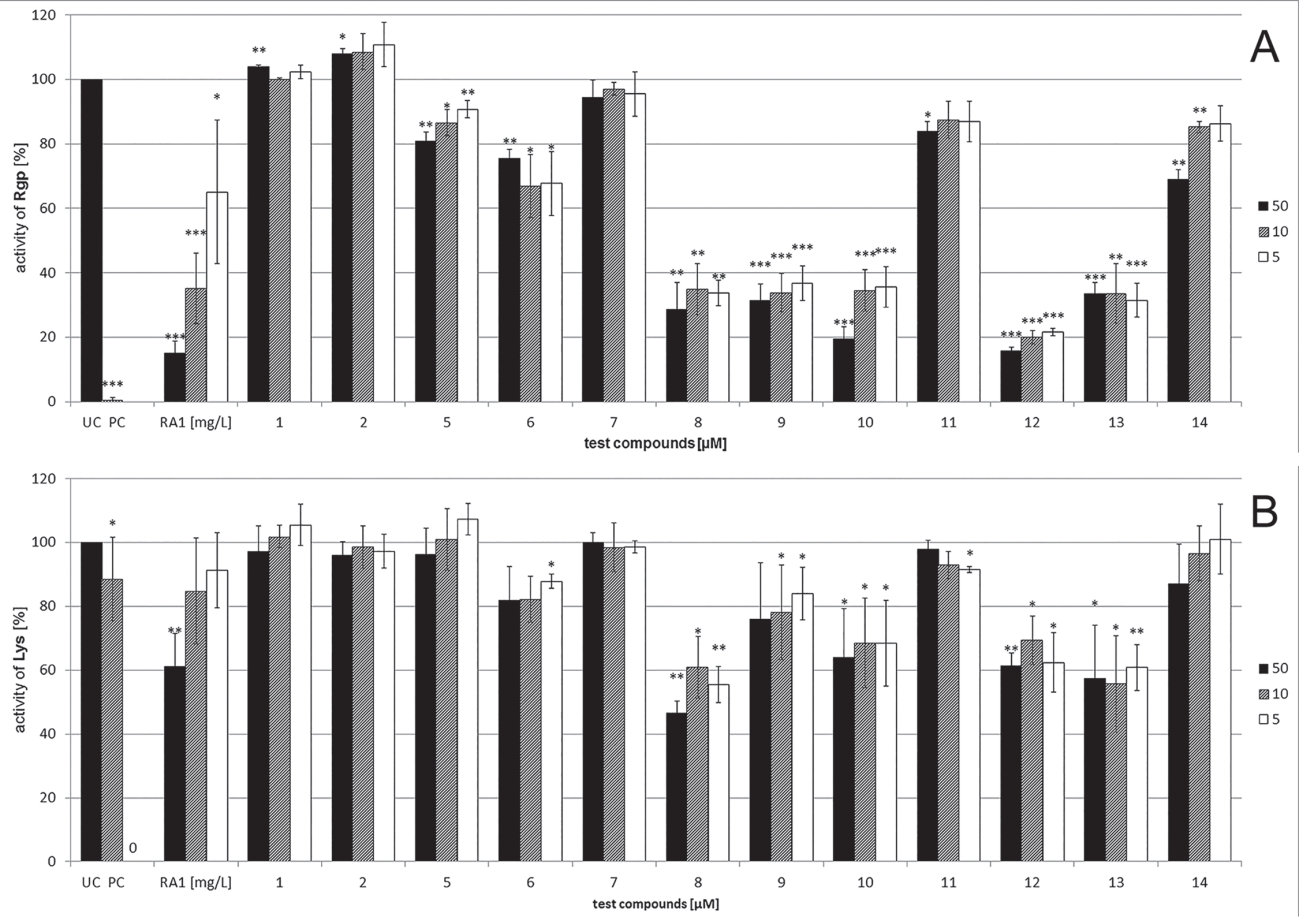
Influence of RA1 (5, 10 and 50 μg/mL) and compounds 1 to 14 (5, 10, 50 μM) on Arg-gingipain (A) and Lys-gingipain (B) protease activity in relation to untreated *P*.*gingivalis*. Leupeptin served as positive control (PC), untreated bacteria as negative control (UC). Data are mean ± SD from 3 independent experiments with n = 5 replicates. * p < 0.05; **: p < 0.01; ***: p < 0.001.

In order to investigate the influence of RA1 on the major adhesins of *P*. *gingivalis*, the arginin- and lysine-gingipain activities were monitored during incubation with RA1 and compounds **1** to **14**. For differentiation of the effects on the Arg- and Lys-gingipain activities, specific fluorescent-labelled peptide substrates were used for protease assays after incubating the bacteria with RA1, namely Bz-Arg-pNA (N_α_-Acetyl-L-lysine-4-nitroanilide) for Arg-gingipain and Ac-Lys-pNA (N_α_-Acetyl-L-lysine-4-nitroanilide) for Lys-gingipain [32]. The tripeptide leupeptide (100 μM), a specific inhibitor of Arg-gingipain, served as positive control (Baba et al, 2001). RA1 inhibited Arg-gingipain in a concentration-dependent manner (Fig. 6). In contrast Lys-gingipain activity was only influenced to a lower extend at the highest concentration. Using flavan-3-ols epicatechin **1** and catechin **2** had no effect on the proteolytic activity of Arg-gingipain, while moderate effects were found for epicatechin-3-O-gallate **5** and epigallocatechin-3-O-gallate **6**. From the dimeric proanthocyanidins procyanidin-B2 7 turned out to be inactive, while the di-galloylated analogon **8** exerted strong protease-inbiting activity; inhibition of Lys-gingipain was observed, but to a much lesser extend compared to the Arg-gingipain. Compound **13**, differing only in the hydroxylation pattern of the B-ring (only 4-hydroxylation) of the upper unit, had the same activity as **8**, being 3,4-dihydroxylated in the B-ring. No differences were observed between the dimeric galloylated 4→8-linked **8**, **13** and the 4→6-linked proanthocyanidins **9**, **10**. Monogalloylation in the lower building block (e.g. in **10**) seemed to be sufficient for activity, while di-galloylation does not seem to be necessary.

Investigation of trimeric procyanidins indicated high activity of the trigalloylated procyanidin **12** while the corresponding ungalloylated compound **11** was inactive. Only moderate activity was found for a mixed A/B-type proanthocyanidin **14**.

Lys-gingipain activity was only influenced to a minor extent by the di- and trimeric galloylated procyanidins.

### RA1 inhibits *P*. *gingivalis* induced hemagglutination

While the previous investigation have shown that RA1 and its polyphenolic compounds interact with the gingipains a potential influence of the extract on *P*. *gingivalis* mediated hemagglutination, induced by fimbrillin (*fimA*) and gingipain was to be investigated by hemagglutination assay. *P*. *gingivalis* were pre-treated for 30 min with different concentrations of RA1 (1 to 1000 μg/mL) and incubated after a serial dilution together with human erythrocytes. As displayed in Table 2 RA1-treated bacteria showed diminished inhibition of hemagglutination.

**Table 2:** Influence of RA1 on *P*. *gingivalis*-mediated hemagglutination.

Concentration RA1 [μg/mL]				
*1000*	*500*	*100*	*50*	*10*
2.5 ± 0	2.0 ± 0	1.5 ± 0	1.0 ± 0	0.5 ± 0

Values from 3 independent experiments display the respective titer shifts related to the untreated control groups.

### RA1 does not influence gene expression of rgpA, kgp and fimA

Inhibition of bacterial adhesion and virulence factors of *P*. *gingivalis* could theoretically result in a specific feed-back reaction of the bacterium, leading to an upregulated expression of the respective proteins. For that RT-PCR studies were performed on the influence of RA1 on the expression of *rgpA* for Arg-gingipain, *kgp* for Lys-gingipain and *fimA* for fimbrillin. Expression rates of the respective genes were constant in *P*. *gingivalis* liquid cultures over incubation periods from 2 to 12 h, as monitored by the corresponding ΔCT values. Treatment of the bacteria with RA1 at 100 μg/mL did not influence the gene expression (data not shown).

### Galloylated procyanidins are predicted in silico to interact with the peptide binding site of Arg-gingipain and hemagglutination domain

**Fig 7:**
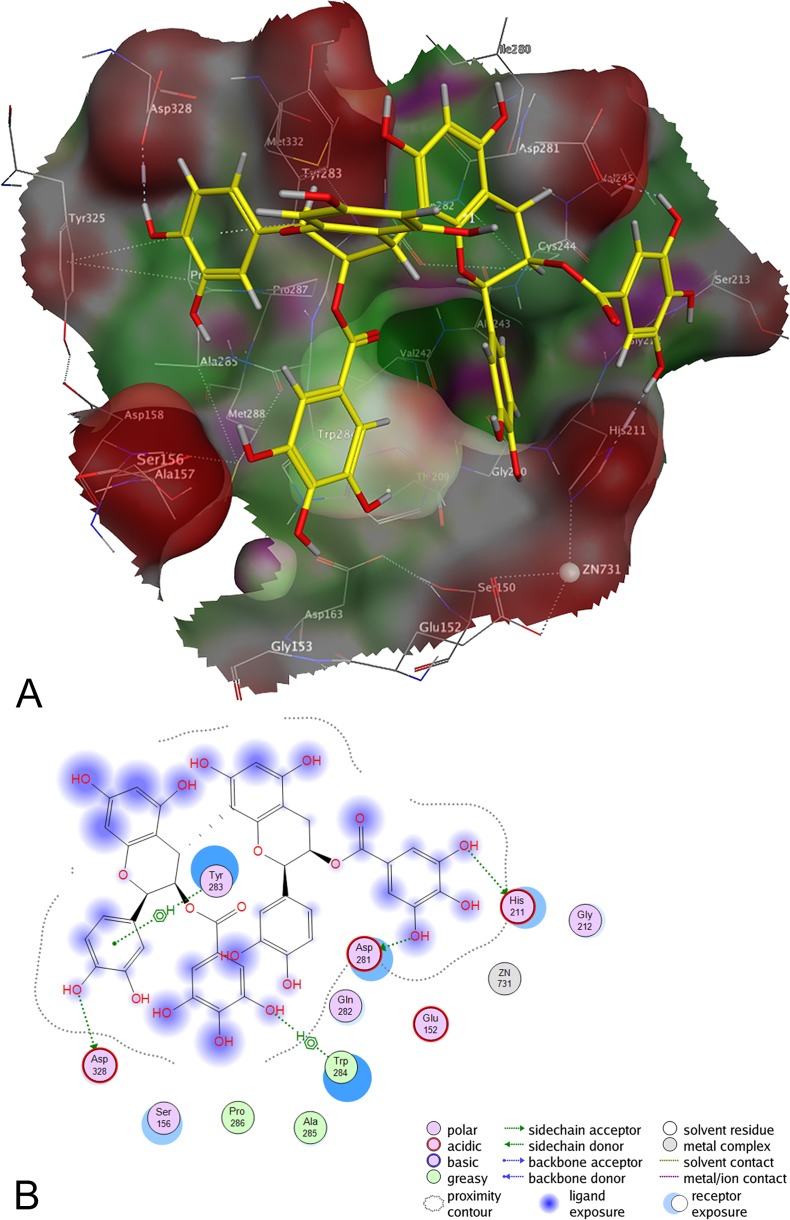
Protein-ligand docking of epicatechin-3-O-gallate-(4β→8)-epicatechin-3-O-gallate 10 into the binding cavity of Arg-gingipain. (**A**) 3D model; protein: green: hydrophobic, purple: polar, red: exposed; ligand: yellow: carbon, light grey: hydrogen, red: oxygen, blue: nitrogen; (**B**) 2D model.

To visualize the binding of components from RA to the Arg-gingipain, 14 selected compounds were docked *in silico* into the active site of Arg-gingipain [42] by means of the software package MOE (Fig. 7). Compound **10** showed the most favorable calculated docking score (S = −6.84 kcal/ml), followed by compound **13**. The data demonstrated a higher score of galloylated compounds in comparison to the unsubstituted oligomeric proanthocyanidins, suggesting a notably stronger anchorage of galloylated molecules in contrast to ungalloylated compounds and offering a straightforward explanation for the strong activity of this digalloylated dimer. Aside from this, the investigated dimers yielded a better docking score than the monomeric flavan-3-ols. These results further corroborate the observation depicted in the functional bioassays: An increase in the degree of polymerization and galloylation enhances the binding of proanthocyanidins. As discussed already above, these results are in contrast to a model favoring the unspecific “coating” of the gingipain by tannin-like polyphenols. The strong anchoring of the galloylated compounds in the active side specifically inhibits the bacterial adsorption process.

### Galloylated procyanidins are predicted in silico to interact with the adhesion domain of Lys-Gingipain

According to the procedure mentioned above for the docking performed on Arg-gingipain all compounds displayed in Fig. 1 were docked into the hemagglutination (HA) domain of Lys-Gingipain (protein database ID “3M1H”) employing the software package MOE. The calculated docking poses (Table 3) also demonstrated a higher docking score of the compounds containing gallic acid moieties just as shown for the Arg-gingipain docking. An increased degree of oligomerization, similar as observed for Arg-gingipain, resulted in an enhanced binding of the proanthocyanidins to HA (Table 3). Additionally the calculated docking scores correlated with the total amount of H-bond-donors and -acceptors present in the examined molecules. This indicates a tannin-like effect of the compounds investigated and therefore suggests an unspecific interaction with the HA domain.

**Table 3:** Docking scores for compounds 1 to 14 from RA1 for the proteolytic domain of Rgp and for the hemaglutinin (HA) domain and correlation of the respective docking scores for Rgp with Arg-gingipain activity as determined within the protease assay (Rgp activity at 50 μM in %, related to the untreated control groups).

Compound	Proteolytic domain		HA domain
	Docking score [kcal/mol)	Rgp activity [%]	Docking score [kcal/mol)
1	−4,44	104	−5,91
2	−4,60	108	−5,55
3	−3,68	n.d.	−6,73
4	−4,28	n.d.	−5,61
5	−4,80	81	−6,96
6	−4,95	76	−7,34
7	−5,77	94	−7,22
8	**−6,11**	29	**−8,53**
9	**−6,44**	31	**−8,19**
10	**−5,91**	19	−8,18
11	−5,36	84	**−8,75**
12	**−6,04**	16	**−8,62**
13	**−6,54**	34	**−8,47**
14	−5,78	69	−7,81

## Discussion

The present study proves the concept that polyphenol enriched plant extracts can strongly interfere with adhesion or invasion of *P*. *gingivalis* to KB cells under *in vitro* and *in situ* conditions. The adhesion assays performed in this study cannot differentiate clearly if RA1 only inhibits the bacterial attachment of *P*. *gingivalis* to the cells or if RA1 also interacts with the invasion into the cell. On the other side confocal laser scanning microscopy clearly indicates that also the bacterial invasion is drastically influenced. On the bacterial side RA1 interacts with surface proteins (e. g. Arg-gingipains, hemagglutinin) and changes functionality and reactivity. Especially the anti-gingipain activity by RA1 seems to be central due to the fact that the gingipain complex is responsible for adhesion to host cells, for conversion of host proteins to bacterial nutrition, but also for maturation of other virulence factors as e.g. *FimA* [25, 47]. Blocking of the bacterial proteases with diminished bacterial adhesion and reduced proteolytic activity has been described for some natural products and synthetic protease inhibitors [14, 48, 49] and also the use of antibodies directed against gingipains [50, 51] led to reduced colonization and pathogenicity of *P*. *gingivalis* in animal infections models.

Interestingly, coincubation assay with bacteria, host cells and RA1 all together, indicates a much stronger antiadhesive activity compared to only preincubation of bacteria with RA1. This might be a hint that also targets at the host cell being influenced and blocked by RA1. As α5β1-integrin is an important receptor for interaction with *FimA* from *P*. *gingivalis* [52] further studies using antibodies against these proteins could help to elucidate the mode of action of RA1.

It seems interesting that inhibition of the Arg-gingipain proteases did not lead to a feed back mechanism of increased gene expression for *rgp*A, which indicates that the gingipain activity is not controlled by bacterial cellular programs. This is in line with recent studies on correlation of *H*. *pylori* adhesion with the gene expression for the respective bacterial adhesins which showed that the adhesion of *H*. *pylori* is not related to changes in gene expression of the adhesins and virulence factors [53, 54].

In the present study the functional characteristics of fimbrial activity has not been assessed. Fimbrillin, the major subunit of the fimbriae, is encoded by the *fim*A gene and displays a molecular mass of approximately 45 kDa [55]. Inactivation of the fimA gene results in decreased attachment to epithelial cells compared to wild-type *P*. *gingivalis* [56]. *Fim*A induces the release of proinflammatory cytokines in macrophages and is also involved in bacteria-induced inflammatory bone desorption [57, 58]. In case of further studies indicating reduced activation of inflammatory cytokines in host cells after treatment of bacteria with RA1 a detailed investigation of the influence of the extract on fimbriae will be promising.

Conclusive specific preventive strategies for early inhibition of periodontitis are still not feasible. Oral hygiene products with antibacterial activity could serve that purpose, but due to the low specificity of those products, both the physiology of the oral microflora can change over the time and host tissues can be harmed by long term use of such biocide products. Antiadhesive compounds with a higher specificity could be an alternative strategy for early prevention of bacterial adhesion to the epithelium of the gingival sulci. Until now only few *in vitro* studies have been performed on this topic, but the published data are promising concerning usage of complex plant extracts enriched with mixtures of polysaccharides and polyphenols as an effective and specific instrument against *P*. *gingivalis* [59, 60, 61].

Due to the fact that *P*. *gingivalis* cell adhesion predominantly relies on the functions of gingipain proteases, the use of antiadhesive compounds influencing protein-protein interactions should be favoured. In contrast, carbohydrate-protein interactions, as known for other adhesive gram-negative bacteria [53, 62, 63] are less important for *P*. *gingivalis* binding to eukaryotic cells. Manipulation of the *P*. *gingivalis* OMP functions can be achieved either by soluble, exogenous modified substrates of the gingipains as e.g. peptides or proteins, or by astringent polyphenols which influence protein structures via H-H-bound interaction (short time contact) or by covalent binding and protein aggregation (longer contact time, oxidative conditions) [64]. The use of polyphenol mixtures should be favoured due to two reasons: i) adstringent affinity of proanthocyanidins to proteins is dependent on structural features, ii) high molecular compounds, especially longer oligomeric or polymeric proanthocyanidins, can exert strong interaction, but have a low solubility. In contrast, low molecular derivatives are perfectly soluble under aqueous conditions, acting very fast against proteins, but have a low adstringency and do not adhere over longer time intervals to proteins. Complex mixtures of polyphenols with different clusters of polymerisation and different substructures also have the advantage of influencing their own solubility, probably by formation of charge-transfer complexes between the different molecules. Therefore standardized proanthocyanidin mixtures are anticipated to be more effective than the isolated and purified compounds, also leading to a multifaceted effect against various surface proteins of the target cells [65]. A clinical correlation between the intake of polyphenol mixtures from aqueous extracts from green tea or from *Vaccinum macrocarpum* (cranberry) and the incidence and severity of periodontitis has recently been documented [66, 67]. Many questions about the properties and functions of *RA1* extracts remain to be answered, especially concerning how specific the galloylated oligomers are really: A recent report of di-galloylated procyanidin B2 indicated inhibitory activity of this compound against viral surface proteins, and galloylated flavan-3-ols from green tea are currently used as in U.S. as FDA-registrated drug against genital warts [68].

At the moment it seems difficult from the toxicological point to assess what consequences it could have if galloylated flavans inhibit so many different molecular targets; on the other side the present study has indicated no signs of cell toxicity within the *in vitro* assays at low concentration and it has to be kept in mind that the use of *R*. *acetosa* as food ingredient in Europa has been widely documented.

The normal benign oral microbiome consists of almost 20.000 different phylotypes that coexist in a well balanced community with the host [69]. In periodontal disease, the composition of this community has been shown to have dramatically changed. This observation could be taken as a sign that specific bacteria that were either not or only barely detectable in health are involved in the pathogenesis of periodontal disease. This hypothesis of a specific polymicrobial etiology has dominated the understanding of the pathogenesis of periodontal disease for almost 4 decades and was considered to be a great obstacle for any specific preventive or therapeutic antimicrobial approach. However, a different composition could also imply that the disease is caused by a dysbiosis of the oral microbiota, i.e. a change in the relative abundance of individual components of the microbiota compared with their abundance in health, leading to alterations in the host-microbial crosstalk sufficient to initiate inflammatory disease [70] This concept is based on the observation that a single pathogen of low abundance can induce pathogenic host-polymicrobial interactions through the normally benign oral microbiota. Specifically in a mouse model, *P*. *gingivalis* at very low colonization levels (<0.01% of the total bacterial count) has been demonstrated to induce periodontal disease accompanied by significant alterations in the number and community organization of the oral commensal bacteria. These microbial changes take place soon after *P*. *gingivalis* colonization and precede the onset of inflammatory bone loss, indicating that the intraoral dysbiosis is the etiologic cause of the disease. The mandatory participation of the commensal microflora in the disease pathogenesis was shown by the failure of *P*. *gingivali*s alone to cause periodontitis in germ-free mice (Low-abundance biofilm species orchestrates inflammatory periodontal disease through the commensal microbiota and complement [71]. The result of these findings demonstrate that *P*. *gingivalis* plays a paramount role in the pathogenesis of periodontal disease. Thus an antimicrobial therapy targeted against *P*. *gingivalis* may result not only in the suppression or eradication of *P*. *gingivalis* but also in the re-establishement of a healthy oral microbiome.

However, the clear economical advantages of such polyphenol-enriched extracts, which can be easily and reliably produced from the plants by a simple process, seem to justify the efforts that have to be taken until clinical usage. Therefore and because of the interesting bioactivity, further developments towards toxicological and clinical testing are promising and should have a high potential.
